# The Willingness of the Healthcare Professionals Working in Healthcare Institutions to Accept the First Dose of COVID-19 Vaccine in Jordan: A National Survey

**DOI:** 10.3390/vaccines10071138

**Published:** 2022-07-17

**Authors:** Mamdouh El-hneiti, Abeer Shaheen, Malakeh Z. Malak, Rawan Al-Hussami, Sakher Salem Al-Hiary, Mutasem Elfalah, Mahmoud Al-Hussami

**Affiliations:** 1 School of Nursing, The University of Jordan, P.O. Box 13807, Amman 11942, Jordan; abeer.shaheen@ju.edu.jo (A.S.); m.alhussami@ju.edu.jo (M.A.-H.); 2 Faculty of Nursing, Al-Zaytoonah University of Jordan, P.O. Box 130, Amman 11733, Jordan; malakeh.m@zuj.edu.jo; 3 Faculty of Medicine, The University of Jordan, P.O. Box 13807, Amman 11942, Jordan; roa0185704@ju.edu.jo (R.A.-H.); m.alrabie@ju.edu.jo (M.E.); 4 Jordan University Hospital, P.O. Box 13046, Amman 11942, Jordan; sakher_horizon@yahoo.com

**Keywords:** acceptance, COVID-19, healthcare professionals, COVID-19 vaccine

## Abstract

Health workers play an important part as role models, advocates for vaccination, vaccinators and educators in a community. Furthermore, they are at high risk of being infected with COVID-19 as they are on the frontlines. Thus, this study purposed to determine the willingness of the healthcare professionals working in healthcare institutions to accept the first dose of COVID-19 vaccine in Jordan. A cross-sectional design and a convenience-sampling method were used to recruit the study population from different healthcare sectors. A web-based survey was used to collect data. Findings showed that 1594 healthcare professionals responded and 74% of them were aged less than 45 years. Almost 65% of the respondents were registered nurses and 68.4% of them were married. A total of 94.9% of the participants heard about the COVID-19 vaccine, but only 56.5% of them had had the opportunity to attend lectures/discussions about COVID-19 vaccine. Official government websites were the primary source of obtaining information about COVID-19 (36.3%). The willingness of acceptance rate of COVID-19 vaccine was 63%. There were positive correlations between vaccine acceptance and compliance toward COVID-19 infection control precautions (*r* = 0.119, *p* < 0.01), knowledge about COVID-19 (*r* = 0.256, *p* < 0.01), age (*r* = 0.170, *p* < 0.01), and years of experience (*r* = 0.105, *p* < 0.01). Furthermore, age, knowledge, and compliance were significant predictors of the acceptance of the COVID-19 vaccine. Thus, it is necessary to develop specific interventions for healthcare professionals with low acceptance rates and take into consideration the predictors of COVID-19 vaccine acceptance. Further research is needed to explore the factors influencing the refusal of the COVID-19 vaccine by healthcare professionals.

## 1. Introduction

Since the appearance of COVID-19 in December 2019, scientists began to develop vaccines to end this pandemic [1]. Vaccines are considered one of the most effective public health measures which save millions of lives yearly [2,3]. Currently, ten vaccines are authorized by at least one national regulatory authority for public use (COVID-19 Vaccine Tracker, 2021). According to the Centers for Disease Control and Prevention (CDC) [4], only Pfizer-BioNTech (BNT162b2) and Moderna (mRNA-1273) vaccines are authorized and recommended to prevent COVID-19 in the U.S.

Global authorities including the World Health Organization (WHO) and CDC have recommended giving COVID-19 vaccines to healthcare personnel; long-term care facility residents; frontline essential workers; people aged 75 years and older; people aged 65 through to 74 years; people aged 16 through to 64 years with underlying medical conditions; and other essential workers [5].

Healthcare professionals play an important part as role models, advocators for vaccination, vaccinators, and educators in a community. Furthermore, they are at high risk to be infected with COVID-19 as they are on the frontlines. It has been confirmed in behavioral insights surveys, for instance, that the family doctor is the most trusted source of information related to COVID-19 [6]. As family doctors are vaccinators themselves, they would play an important role as advisors of vaccination for all other groups [6]. A systematic review was conducted in Jordan and other Middle East countries by Mahmud et al. [7] which reported that psychosocial problems including depression, anxiety, stress, and insomnia among healthcare professionals when they care for patients diagnosed with COVID-19 might be the reason for the reluctance of healthcare professionals to take the COVID-19 vaccine. Globally, a systematic review reported vaccine acceptance varied widely and ranged from 27.7% to 77% among physicians and nurses; additionally, vaccine hesitancy was a common factor among healthcare professionals [8]. Another systematic review demonstrated that the healthcare professionals endorsed moderate acceptance of COVID 19-vaccination, in which 51% reported willingness to get vaccinated [9]. Additionally, a previous Jordanian study conducted among young adults demonstrated that one-fifth (19.9%) of the participants were willing to administer COVID-19 vaccinations [10]. However, willingness of healthcare professionals to accept the first dose of COVID-19 vaccines has not been examined adequately in international and Arab countries including Jordan. Therefore, this study could help develop the interventions and strategies that might improve willingness for these vaccinations among healthcare professionals who are on the frontlines of providing healthcare, which could reflect on improving acceptance of COVID-19 among healthcare professionals. Thus, this study purposed to determine the willingness of the healthcare professionals working in hospitals to accept the first dose of the COVID-19 vaccine in Jordan. Additionally, the study has the following research questions:

1. What is the willingness of acceptance level of the first dose of COVID-19 vaccine among the healthcare professionals working in healthcare institutions in Jordan?
2. What are the relationships between the willingness of acceptance of the first dose of the COVID-19 vaccine; knowledge of COVID-19; compliance of infection control precautions (ICPs); and sociodemographic factors among the healthcare professionals working in healthcare institutions in Jordan?
3. What are the significant factors influencing the willingness of acceptance of the first dose of the COVID-19 vaccine among the healthcare professionals working in healthcare institutions in Jordan?

## 2. Methods

### 2.1. Design

This study used a cross-sectional design to administer a self-reported online questionnaire. Data were collected over four weeks from 3 January to 15 February 2021.

### 2.2. Study Settings

Jordan is one of the 23 Arab countries in the Middle East bordered by Saudi Arabia to the south and the east, Iraq to the northeast, Syria to the north and Palestine to the west. The first-level subdivision in Jordan is the governorate [11] (United Nations 2017). The governorates are divided into districts, which are often further subdivided into subdistricts. In addition, Jordan is divided into 12 governorates; Amman Governorate has the largest population among them as well as the country’s economic, political and cultural centers [11] (United Nations 2017). The health services in Jordan comprise multiple providers, namely: public sector; private sector; and Royal Medical Services (RMS). The study settings were selected from the healthcare institutions according to large occupancy and included acute care wards (i.e., medical and surgical) and out-patient departments of general hospitals (i.e., public sector, private sector, and RMS).

### 2.3. Sampling

The reference population were all healthcare professionals including registered nurses, physicians, pharmacists, lab technicians, and dentists who were working in different healthcare sectors with a total number of around 22,000 healthcare workers [12]. However, the accessible population for this study were all healthcare professionals who were willing to complete the survey questionnaire online in the selected institutions. Convenience and snowball sampling methods were used to recruit the study population from different healthcare sectors in the selected settings. The inclusion criteria included healthcare professionals who were working in a healthcare facility (i.e., public, private, or RMS).

The estimated sample size was calculated using the software computer program G*power [13]. Using F tests—ANOVA: One-Way ANOVA, between-groups with a medium-effect size of 0.15, α significance error of 0.05, level of power 95%, and number of groups = 5, a minimum of 1594 was needed to conduct this study.

### 2.4. Ethical Considerations

Ethical approval was obtained from the Scientific Research Committee at Al- Zaytonnah University of Jordan (Approval No. 119-2019/2022). The questionnaire was provided in the first page with instructions regarding the purposes of the study and instructions about how to answer questions. In order to maintain anonymity and confidentiality, the survey avoided mentioning any personal information of the participants such as names, address, and any contact details. Access to the survey forms was possible only through password-protected login of the researchers. Additionally, e-written informed consent was included as a part of this survey.

### 2.5. Measurements

The data were collected using a self-structured questionnaire. This questionnaire consisted of a 23-item survey instrument, which was adapted by the authors using WHO course materials [14] on emerging respiratory viruses, including COVID-19, and covered the domains of healthcare professionals’ characteristics, knowledge, compliance related to COVID-19, and acceptance of COVID-19 vaccine. Moreover, demographic details and work characteristics information (8 items) were added. Knowledge of COVID-19 vaccine was measured using five items that were answered as yes, no, and do not know. The overall score for knowledge ranged from 0 to 5; each correct answer was given a point toward the final score. Compliance toward COVID-19 infection control was evaluated using nine statements rated on a 4-point Likert scale ranging from 1 = strongly disagree to 4 = strongly agree, and the score for compliance ranged from 9 to 36. Intention to receive a COVID-19 vaccine was measured using one item rated on a four-point scale (‘definitely not’ to ‘definitely yes’).

This survey was translated from English to Arabic by English translator, then back-translated to English to ensure its accuracy. Additionally, the Arabic-translated version was checked by an Arabic translator who has a Ph.D. in Arabic language. Modifications were applied to adjust the questionnaire based on the Jordanian context. Then, the content validity index (CVI) for the Arabic version of the survey was evaluated by five experts in the domain of a study. After returning the experts’ responses, they agreed that the measurements were valid. The overall content validity of this instrument was 88.6%, which is considered acceptable.

The pilot study was performed to ensure that all statements are clear and understandable, and to determine the time required to complete the questionnaire. The healthcare professionals (*n* = 15) were chosen to perform this piloting study. Findings demonstrated that all statements were clear, straightforward, and understandable. Furthermore, the required time for completing a questionnaire was 10–15 min.

The internal consistency of reliability was assessed using Cronbach’s alpha; the results for the total scale obtained from the pilot study was 0.72. Additionally, Cronbach’s alpha for the three dimensions of the questionnaire was as follows: knowledge = 0.71, compliance = 0.87, and acceptance = 0.68.

### 2.6. Data Collection Procedure

At the beginning, contact with the managers of healthcare institutions was conducted to facilitate the data collection procedure. We used Qualtricst, which is an online survey software program to create the survey questions. The data were collected, in which the online survey was launched using the ‘G-mail Google’ platform, and the link to a Google form was shared to all WhatsApp groups which were created by these healthcare institutions for healthcare professionals. The survey form was displayed on scrollable pages in which a participant can move back and forth with the survey. This survey link was pilot-tested for usability and technical functionality with 20 healthcare professionals who met the inclusion criteria for the study participants before sharing, and the data collected from this pilot testing were not included in the final report of this study.

All the items were marked as compulsory and, thereby, all submitted forms were complete. Furthermore, each participant had one chance to submit the survey and a second trial was automatically denied. Data were collected over four weeks from 3 January to 15 February 2021.

### 2.7. Data Analysis Plan

The data were entered, cleaned, screened for missing items, and analyzed using Statistical Package for the Social Sciences (SPSS, version 24, IBM, Armonk, NY, USA). Descriptive statistics (frequency, percentages, means, and standard deviations) were used to describe the basic features of the data in a study by providing simple summaries about the sample and the measures. In addition, inferential statistics were used (correlation coefficient and multiple regression) to examine the relationship between study variables and the factors influencing the acceptance of COVID-19 vaccine. The significance of the results was set at *p* ≤ 0.05. Data were checked for the presence of outliers, normality, linearity, homoscedasticity, and multicollinearity. Additionally, internal consistency was checked using Cronbach’s alpha coefficient. The variables in this study are quantitative with skewness ranging from 0.06 to 0.071, which is acceptable to say that it is approximately normally distributed. Using Cook’s distance examination, outliers were studied. As Cook’s values for all products were less than 1, where the highest value found was 0.04, no outlier was found. Additionally, multicollinearity was confirmed through checking the values of the correlation coefficient (*r*) between the predictors, and it was observed that it did not exceed 0.70, which indicates that the predictors chosen were measuring different things and there was an absence of multicollinearity.

## 3. Results

From a total of 2147 invitees, 1594 healthcare professionals were included in the final analysis and completed the study questionnaire with a response rate of 74.3%. Table 1 displays the descriptive statistics of the demographic variables of the study participants. Of the 1594 participants, 1036 (65%) were female and 559 (35%) were male.

Most of the respondents (74%) were below 45 years of age. The majority of respondents were registered nurses (*n* = 991, 65.2%), and 68.4% (*n* = 1090) of them were married. The majority (94.9%) of the participants reported that they heard about the COVID-19 vaccine, but only 56.5% of them had had the opportunity to attend lectures/discussions about the COVID-19 vaccine. Moreover, the primary source of information that they obtained about COVID-19 was through official government websites (36.3%) (see Table 1).

Bivariate associations were performed between knowledge mean score, compliance mean score, age of respondents, years of experience, and intent to accept the COVID-19 vaccine among healthcare professionals in Jordan. When respondents were asked, ‘will you agree to vaccinate against COVID-19?’, of the 1594 respondents, 327 (20.5%) chose strongly agree to accept taking the hypothetical vaccine, 677 (42.5%) chose agree to accept taking the hypothetical vaccine, 366 (23%) chose disagree, and 157 (9.8%) chose strongly disagree to accept taking the vaccine, while 67 (4.2%) respondents did not record their answer. Hence, the acceptance rate of the COVID-19 vaccine was 63% (*n* = 1004) and 32.8% (*n* = 523) reported no acceptance of the vaccine (see Table 2).

Pearson’s correlation was calculated to find any significant relationship with *p* values less than 0.05. The results revealed that there were positive correlations between vaccine acceptance and compliance mean score toward COVID-19 ICPs (*r* = 0.119, *p* < 0.01) and between vaccine acceptance and knowledge mean score regarding COVID-19 (*r* = 0.256, *p* < 0.01). In addition, the result showed a positive relationship between age and years of experience with vaccine acceptance (*r* = 0.170, *p* < 0.01; *r* = 0.105, *p* < 0.01, respectively). Furthermore, the relationship between the compliance, age, and years of experience was found statistically significant (*r* = 0.280, *p* < 0.01; *r* = 0.129, *p* < 0.01, respectively). Finally, the results revealed a statistically significant relationship between healthcare professionals’ (HCPs) knowledge towards COVID-19, age, and years of experience (*r* = 0.115, *p* < 0.01; *r* = 0.129, *p* < 0.01, respectively). However, the result revealed a strong positive correlation between years of experience and age of healthcare professionals (*r* = 0.87, *p* < 0.01), as shown in Table 3.

Hierarchical multiple regression analysis was used to find the factors that are significantly affecting acceptance of the COVID-19 vaccine. The results showed that model one that contained age explained 7.4% (*R*^2^ = 0.074) of the variance in accepted COVID-19 vaccine (Table 3) and the model was significant (*p* ≤ 0.05). After entry of years of experience in the second model, the total variance explained by the model was 7% (*R*^2^ = 0.079) and was not significant (*p* = 0.21). The third model, by adding (knowledge towards COVID-19), accounted for an additional 24% of the variance in compliance, (*R*^2^ = 0.246) which was statistically significant (*p* ≤ 0.001). The fourth model (compliance towards COVID-19 safety measures) was entered on the last step, and it accounted for an additional 8% of the variance in compliance (*R*^2^ = 0.08) which was statistically significant (*p* ≤ 0.001). The variables in the first model, third model, and the fourth model accounted for 40% of the variance in overall acceptance of the COVID-19 vaccine. The result revealed that age, knowledge, and compliance accounted for significant predictors of the acceptance of the COVID-19 vaccine. It is noted also that participants’ years of experience appeared to be a nonsignificant factor in model two.

COVID-19 is considered a major global public health crisis that has resulted in 152,888 infections and 1413 deaths in healthcare professionals. Compared to other world regions such as Africa and Europe, the Eastern Mediterranean region had the highest number of reported deaths per 100 infections [15]. Previous literature suggested that public health workers are experiencing workload changes because of their role in preventing and controlling the spread of COVID-19. They are also experiencing lifestyle changes during the COVID-19 pandemic such as physical inactivity and daily sweet food consumption [16]. Healthcare professionals are more likely to have access to the vaccine at an early stage and play a significant role in providing information about the vaccine for the general population. It is crucial to vaccinate the maximum number of healthcare professionals to prevent the infection and the loss of our frontline workforce that already suffers from a serious shortage. Thus, it is important to assess predictors of vaccine acceptance among healthcare professionals to help healthcare institutions and policymakers increase the uptake of the vaccine. This study investigated the willingness of the acceptance rate of the COVID-19 vaccine and factors that affected the willingness of the acceptance of the COVID-19 vaccine among healthcare professionals in Jordan.

The willingness of acceptance rate of the COVID-19 vaccine in the current study was 63%. In comparison with a U.S. study, 36% of the healthcare professionals were willing to take the COVID-19 vaccine while 56% were not sure or would wait to review more data [17]. Another study in the Democratic Republic of the Congo found that 27.7% of healthcare professionals were willing to receive a COVID-19 vaccine [18]. On the contrary, the reported willingness for vaccine acceptance rate among healthcare professionals in France and China was 76.9% and 76.4%, respectively [19,20]. The willingness for vaccine acceptance rate in this study was higher than in the U.S. and Congo but lower than in France and China. Additionally, the willingness of vaccine acceptance rate in this study was higher than the COVID-19 vaccine acceptance among the general public in Jordan, which was 28.4% [21]. The high percentage of the willingness of accepting to take the vaccine among the respondents in our study may highlight the importance of knowledge about the COVID-19 vaccine, as most of the respondents in our study reported that they had heard about the COVID-19 vaccine. In addition, more than half of them attended lectures or discussions about the COVID-19 vaccine, and one-third obtained information about COVID-19 through official government websites. The high willingness of accepting to take the COVID-19 vaccine among HCWs in Jordan may have consequences on the Jordanian community since various studies have shown that vaccinated healthcare professionals are more likely to recommend vaccines to their families and patients [22,23].

The willingness for COVID-19 vaccine acceptance escalated with increasing age and knowledge of COVID-19. Studies that included both the general population and healthcare professional samples indicated that willingness to accept the COVID-19 vaccine was higher among older adults compared to younger adults [17,19,23,24,25]. Age is considered a strong risk factor for the severity of COVID-19 disease, ICU admission rate, and death [26]. Thus, older adults need to be vaccinated as soon as possible and this could explain the increase in the acceptance of the COVID-19 vaccine among older healthcare professionals. In this study, it is noted that respondents relied on official government websites, news media, and social media, respectively, to obtain information about COVID-19. A different pattern was observed in another study among healthcare professionals in Congo in which respondents relied on social media, family, and friends for information on COVID-19 [18]. Similar findings were reported in a global cross-sectional study in which respondents relied on information from official and government websites, news media, and social media to obtain information about COVID-19 [27]. The same study also linked the knowledge about COVID-19 from trustful resources such as government sources with higher vaccine acceptability. These results stress the role of media coverage using different channels to provide healthcare professionals with updated information about issues related to COVID-19. Additionally, this study found that COVID-19 vaccine acceptance increased with increasing compliance toward COVID-19 ICPs among healthcare professionals. This finding implied that healthcare professionals in Jordan did not consider their adherence to ICPs as a substitute for the COVID-19 vaccine. Similarly, a UK study among adults revealed that one of the predictors of COVID-19 vaccine refusal was poor adherence to COVID-19 government guidelines [28]. Inconsistent results were reported by a study conducted in China, which found that an increase in personal protection behaviors decrease vaccine acceptance [29]. A national study found that the main factors for refusal were concerns about the use of these vaccines and loss of trust in them [30,31].

## 4. Strength and Limitations

A large sample of healthcare professionals was surveyed. However, the study had some limitations that should be acknowledged. Firstly, a web-based self-administered survey that was based on participants’ opinions was used, which may affect the responses. Selection bias could also arise as only participants who have access to the internet participated in the study. Secondly, the study sample was convenient and may limit the external validity of findings reported due to selection bias. In particular, the majority of the participants were disproportionately skewed to a group of healthcare professionals (nurses). Furthermore, the study used a cross-sectional design which limits the ability to make a causal inference between study variables. Future studies using a qualitative design and a more representative sample of healthcare professionals are recommended to enhance our understanding of this issue.

## 5. Conclusions

The willingness to accept the COVID-19 vaccine among healthcare professionals in Jordan was high, with a large percentage of healthcare professionals accepting to take the vaccine. Determinants of willingness of vaccine acceptance were older age, knowledge of the COVID-19, and compliance with the COVID-19 ICPs.

## 6. Recommendations

It is crucial to measure healthcare professionals’ acceptance of COVID-19 vaccine and report it to the leaders. Further research is needed to understand the factors influencing the refusal of the COVID-19 vaccine by healthcare professionals to improve the acceptance rate. Healthcare professionals who are not willing to take the COVID-19 vaccine should be targeted. Specific interventions should be developed to enhance acceptance among this specific cohort. These interventions should take into consideration the predictors of COVID-19 vaccine acceptance. The results of this study emphasize the importance of providing healthcare professionals with updated information related to the COVID-19 vaccine through different channels such as lectures, discussions, and official government websites. It is also important to clarify to healthcare professionals that adherence to international precautions is not a substitute for the COVID-19 vaccine.

## Figures and Tables

**Table 1:** Sociodemographic characteristics of healthcare workers (*n* = 1594).

Characteristics	*n* (%)
**Gender**	
Male	559 (35)
Female	1036 (65)
**Age**	
<25 years	80 (5.0)
25–34 years	720 (45.2)
35–44 years	380 (23.8)
45–54 years	289 (18.2)
≥55 years	125 (7.8)
**Marital Status**	
Single	365 (22.9)
Married	1090(68.4)
Divorced	104 (6.6)
Widow	35 (2.1)
**Occupation**	
Registered nurse	991 (65.2)
Physician	380 (21.6)
Pharmacist	118 (6.9)
Lab technician	55 (2.9)
Dentist	61 (3.3)
**Experience**	
≤4 years	292 (18.3)
5–9 years	400 (25.1)
10–14 years	313 (19.6)
15–19 years	305 (19.2)
20–24 years	187 (11.7)
≥25 years	97 (6.1)
**Source of Knowledge**	
Family and friends	60 (3.8)
News media	533 (33.4)
Official government websites	579 (36.3)
Social media	422 (26.5)
**Heard about COVID-19**	
Yes	1513 (94.9)
No	81 (5.1)
**Attended Lectures/Discussion about COVID-19**	
Yes	901 (56.5)
No	693 (43.5)

*n*: number; %: percentage.

**Table 2:** Willingness of healthcare professionals to accept taking the hypothetical vaccine.

Job Title	Disagree to Receive Vaccine	Agree to Receive Vaccine	Total
Dentist	18	33	51
Lab Technician	22	23	45
Pharmacist	27	78	105
Medical Doctor	109	221	330
Registered Nurse	347	649	996
Total	523	1004	1527

**Table 3:** Pearson’s correlation between knowledge of COVID-19, compliance of infection control precautions, age, years of experience, and willingness of acceptance of COVID-19 vaccine.

Variable	Willingness of Vaccine Acceptance	Compliance	Knowledge	Age	Experience
**Vaccine Acceptance**	1				
**Compliance**	0.119 **	1			
**Knowledge**	0.256 **	0.241 **	1		
**Age**	0.170 **	0.280 **	0.115 **	1	
**Experience**	0.105 **	0.320 **	0.129 **	0.87 **	1

Note. *n* = 1594; ** Correlation is significant at the 0.001 level (2-tailed).

## Data Availability

The data presented in this study are available on request from the corresponding author.
